# Real-Time Seam Extraction Using Laser Vision Sensing: Hybrid Approach with Dynamic ROI and Optimized RANSAC

**DOI:** 10.3390/s25113268

**Published:** 2025-05-22

**Authors:** Guojun Chen, Yanduo Zhang, Yuming Ai, Baocheng Yu, Wenxia Xu

**Affiliations:** 1 School of Computer Science and Engineering, Wuhan Institute of Technology, Wuhan 430205, China; 2 Hubei Engineering Research Center for Intelligent Production Line Systems, Wuhan Institute of Technology, Wuhan 430205, China

**Keywords:** laser vision, dynamic ROI, optimized RANSAC, weld seam extraction

## Abstract

Laser vision sensors for weld seam extraction face critical challenges due to arc light and spatter interference in welding environments. This paper presents a real-time weld seam extraction method. The proposed framework enhances robustness through the sequential processing of historical frame data. First, an initial noise-free laser stripe image of the weld seam is acquired prior to arc ignition, from which the laser stripe region and slope characteristics are extracted. Subsequently, during welding, a dynamic region of interest (ROI) is generated for the current frame based on the preceding frame, effectively suppressing spatter and arc interference. Within the ROI, adaptive Otsu thresholding segmentation and morphological filtering are applied to isolate the laser stripe. An optimized RANSAC algorithm, incorporating slope constraints derived from historical frames, is then employed to achieve robust laser stripe fitting. The geometric center coordinates of the weld seam are derived through the rigorous analysis of the optimized laser stripe profile. Experimental results from various types of weld seam extraction validated the accuracy and real-time performance of the proposed method.

## 1. Introduction

In modern industrial manufacturing, welding is a crucial method for joining and processing materials, extensively used in the aerospace, shipbuilding, automotive, and construction industries [1,2,3,4]. The hazards of the intense arc radiation, high-decibel noise, and toxic fumes generated during welding processes severely constrain the quality consistency and operational efficiency of manual welding. With the advancement of robotic control technology, automated welding has become an inevitable trend in industrial manufacturing. Currently, the mainstream technical approaches can be categorized into two methods: The first method is the teaching-and-playback approach, which simply replicates predefined trajectories. When there are deviations in the workpiece positioning or environmental parameters [5,6], weld seam displacement can occur, resulting in compromised welding quality. The second method involves intelligent welding systems that utilize multi-modal sensing technology. These systems achieve environmental perception and dynamic trajectory compensation using sensor arrays and adaptive control algorithms, and they now represent the core focus of research on automated welding. Different sensing systems, such as visual sensing, acoustic sensing, ultrasonic sensing, and arc sensing [1,5,7,8,9], can be used in this method. The successful implementation of this framework relies on the high-precision, real-time identification of weld seam characteristics. Laser vision sensing technology, with its non-contact measurements, high accuracy, and real-time detection capabilities [5,10,11], has become an essential tool for weld seam detection. However, the complexity and variability of industrial environments, unstable lighting conditions, potential oil stains on workpiece surfaces [12], and disturbances from arc light and spatter during welding often degrade the quality of weld images, posing significant challenges to accurate weld seam extraction.

Currently, methods for weld seam extraction can be categorized into two main approaches: traditional digital image processing techniques and deep learning-based intelligent detection methods [13]. Johan, N. F. et al. [14] achieved the precise extraction of weld seam feature points through three crucial stages: laser extraction, broken-line fitting, and pixel location determination. Wu et al. [15] employed median filtering and the Otsu algorithm for image preprocessing and binarization, combined with an improved Hough transform algorithm to detect the weld seam positions. Muhammad et al. [16] employed median filtering, color processing, and blob analysis to extract weld seam feature points. Li et al. [17] developed various laser stripe templates and similarity evaluation functions, using template matching to extract weld seams in noisy environments. Shao et al. [18] utilized static ROIs for extracting narrow-gap butt welds, but these static ROIs lacked flexibility and were inadequate for complex welding environments. Yu et al. [19] designed a weld tracking algorithm that combines morphological extraction and kernel correlation filtering, achieving the accurate tracking of multiple weld seams.

In recent years, the applications of deep learning in computer vision have attracted significant attention, including YOLOv5, Faster R-CNN, DeepLabV3, etc. YOLOv5 (You Only Look Once version 5) is a state-of-the-art, single-stage object detection model renowned for its speed, accuracy, and ease of deployment. Building on the YOLO architecture, it employs a CSPDarknet53 backbone for feature extraction and PANet for multi-scale feature fusion, enabling the efficient detection of objects across varying sizes. Li et al. [20] employed Mask R-CNN for the instance segmentation of weld seams and integrated Hough transformbased image processing to achieve the high-precision extraction of weld seam trajectories. Gao et al. [21] introduced the RepVGG network and a Normalized Attention Module (NAM) to optimize YOLOv5, enhancing the detection speed for weld seam feature points and enabling accurate extraction with complex backgrounds. Mobaraki, M. et al. [22] implemented an automatic tracking system for fillet welds between pipes and flanges during Gas Metal Arc Welding (GMAW) using a network architecture based on ResNet 101 and the Stacked Hourglass model, which reduced welding defects and the need for rework, saving significant manufacturing costs. Kang et al. [23] replaced the backbone network of DeepLabV3+ with MobileNetV2 and introduced a DenseASPP structure alongside an attention mechanism, specifically focused on laser stripe edge extraction, thereby obtaining clearer laser stripe images and reducing noise interference. Lin et al. [24] proposed a hybrid methodology that initially localizes the welding torch through a YOLOv5-based object detection algorithm. Subsequently, an adaptive ROI-driven image processing algorithm is employed to extract the weld seam centerline, fulfilling the real-time performance and precision requirements of the K-TIG seam tracking system. These studies highlight the robust capabilities of deep learning in complex scenarios. However, deep learning-based methods require extensive amounts of annotated data and offline training, raising the barrier to user adoption. Coupled with high computational complexity, this means that these approaches struggle to meet the stringent real-time demands in welding applications.

To address these challenges, this paper proposes a laser vision-based weld seam extraction method that combines a dynamic ROI and an optimized random sample consensus (RANSAC) algorithm. The method begins by automatically acquiring the laser stripe region using a dynamic ROI. Next, adaptive threshold segmentation is applied to extract the laser stripe, followed by morphological filtering and medial axis transformation for refinement. Finally, an optimized RANSAC algorithm is employed to fit the extracted laser stripe, enabling the precise extraction of weld seam feature points. The main contributions of this study include the following:

- A Dynamic ROI Transmission Mechanism: An adaptive ROI generation strategy based on the geometric features of laser stripes from historical frames is proposed, which intelligently suppresses noise regions through inter-frame spatial correlation analysis.
- Slope-Constrained RANSAC Optimization: The conventional RANSAC algorithm is enhanced by integrating historical slope constraints, where slope threshold restrictions are imposed on random sampling processes to accelerate iterative convergence.
- A Sequential Processing Architecture: A recursive workflow incorporating “pre-arc baseline initialization → dynamic ROI updating → constrained fitting” is established, thereby achieving high-efficiency, real-time tracking throughout the entire welding process cycle.

The structure of this paper is organized as follows: Section 2 elaborates on the proposed methodology. Section 3 details the experimental design and implementation. Section 4 provides a discussion of the experimental results. Finally, Section 5 concludes the paper and outlines potential directions for future research.

## 2. Methodology

### 2.1. System Architecture and Data Acquisition

**Figure 1:**
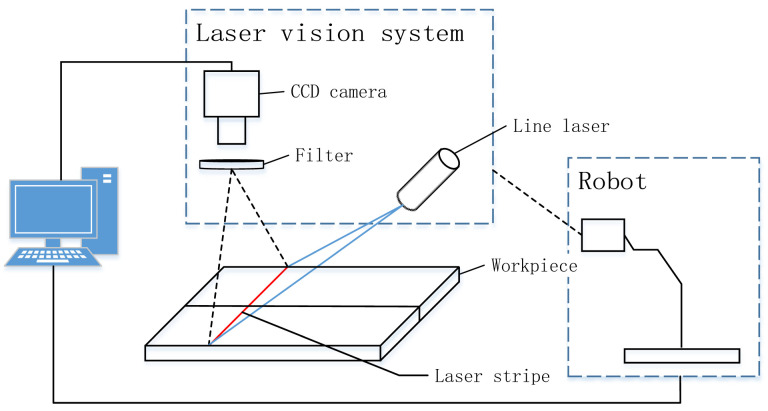
System architecture composition.

The system primarily consists of a welding robot, laser vision system, and computer, as shown in Figure 1. The laser vision system is composed of a filter, line laser, and industrial CCD camera, all of which are encapsulated in a protective shell. The shell and welding torch are mounted on the end of the robot arm, and the filter is installed in front of the CCD camera to effectively filter the arc light generated during welding, reducing the interference from arc light and spatter during image processing.

The laser emitted by the line laser illuminates the surface of the welding workpiece, forming a laser stripe at the point of intersection. The industrial CCD camera captures the weld images with laser stripes. The captured images are transmitted to the computer via TCP/IP communication. The computer then runs the proposed algorithm to extract the weld seam.

### 2.2. Proposed Algorithm

**Figure 2:**
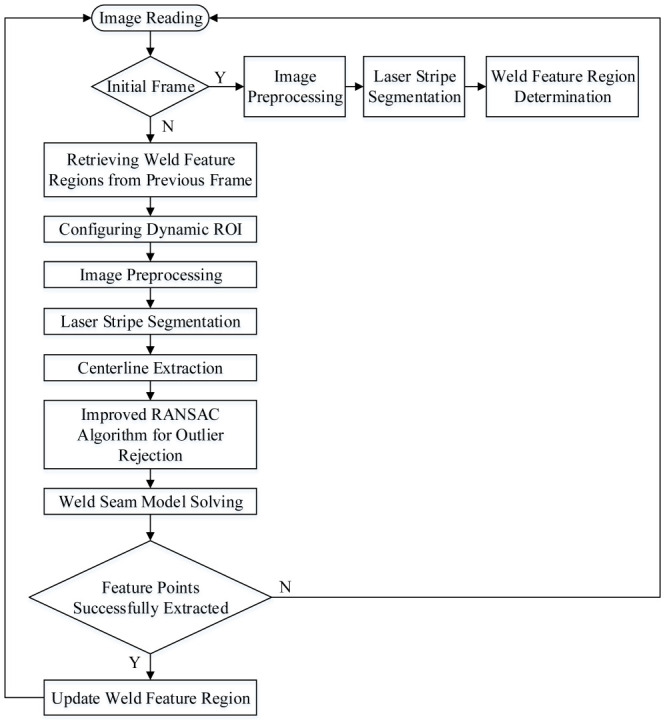
Weld seam extraction process.

During real-time weld seam tracking, the weld trajectory and vision sensor data are acquired continuously. Due to the minimal inter-frame variation between consecutive frames, the recognition results from the previous frame can be leveraged to optimize the processing of the current frame. The detailed process is illustrated in Figure 2.

#### 2.2.1. Dynamic ROI

In weld seam imagery, laser stripe feature regions typically occupy only a small portion of the original image space. Employing a full-frame image processing strategy not only significantly increases the computational load but also degrades the feature extraction accuracy due to arc light interference and spatter noise in highly dynamic welding environments. We propose a temporally correlated dynamic region of interest (ROI) generation method. By analyzing the spatial continuity of weld trajectories and the temporal continuity of image acquisition, the approach constructs the ROI model for the current frame using the laser stripe feature point set from the preceding frame.

Prior to torch ignition at the welding start point, a pristine weld seam image is acquired as the initial reference frame. The subsequent processing of this initial frame extracts the laser stripe profile, mathematically represented as the discrete point set $P^{\left(0\right)}=\left\{p_{i}^{\left(0\right)}\left(u,v\right)|i\in \left[1,N\right]\right\}$, where *N* is the total number of contour pixel points. Through laser vision sensing, the captured weld seam profile can be reduced to three critical geometric feature points [19] along the laser stripe, denoted as $\left\{p_{l}^{\left(t\right)},p_{o}^{\left(t\right)},p_{r}^{\left(t\right)}\right\}$, where *t* represents the frame index, with the determination method explicitly defined as follows:

(1) $$\left\{\begin{array}{c} p_{l}^{\left(t\right)}=p_{i^{*}}^{\left(t\right)},\;\mathrm{where}\;i^{*}=\underset{i}{\arg\min}\;p_{i}^{\left(t\right)}\left(v\right),\;i=1,2,\ldots ,N\\ p_{o}^{\left(t\right)}=p_{i^{*}}^{\left(t\right)},\;\mathrm{where}\;i^{*}=\underset{i}{\arg\max}\;d_{i}^{\left(t\right)},\;i=1,2,\ldots ,N\\ p_{r}^{\left(t\right)}=p_{i^{*}}^{\left(t\right)},\;\mathrm{where}\;i^{*}=\underset{i}{\arg\max}\;p_{i}^{\left(t\right)}\left(v\right),\;i=1,2,\ldots ,N \end{array}\right.$$

As shown in Equation (1), the distance parameter ${d_{i}}^{\left(t\right)}$ is defined as the perpendicular distance from an arbitrary point, ${p_{i}}^{\left(t\right)}$, within the discrete laser stripe contour point set $P^{\left(t\right)}=\left\{p_{i}^{\left(t\right)}\left(u,v\right)\right\}$ extracted from the *t*th frame weld seam image to the segment determined by boundary feature points ${p_{l}}^{\left(t\right)}$ and ${p_{r}}^{\left(t\right)}$.

(2) $$d_{i}^{\left(t\right)}=\frac{A^{\left(t\right)}p_{i}^{\left(t\right)}\left(u\right)+B^{\left(t\right)}p_{i}^{\left(t\right)}\left(v\right)+C^{\left(t\right)}}{\sqrt{{\left(A^{\left(t\right)}\right)}^{2}+{\left(B^{\left(t\right)}\right)}^{2}}}$$

where $A^{\left(t\right)}$, $B^{\left(t\right)}$, and $C^{\left(t\right)}$ are the coefficients of the linear equation corresponding to the line containing segment $p_{l}^{\left(t\right)}p_{r}^{\left(t\right)}$. The equation of the line segment $p_{l}^{\left(t\right)}p_{r}^{\left(t\right)}$ is given as follows:

(3) $$A^{\left(t\right)}u+B^{\left(t\right)}v+C^{\left(t\right)}=0$$

**Figure 3:**
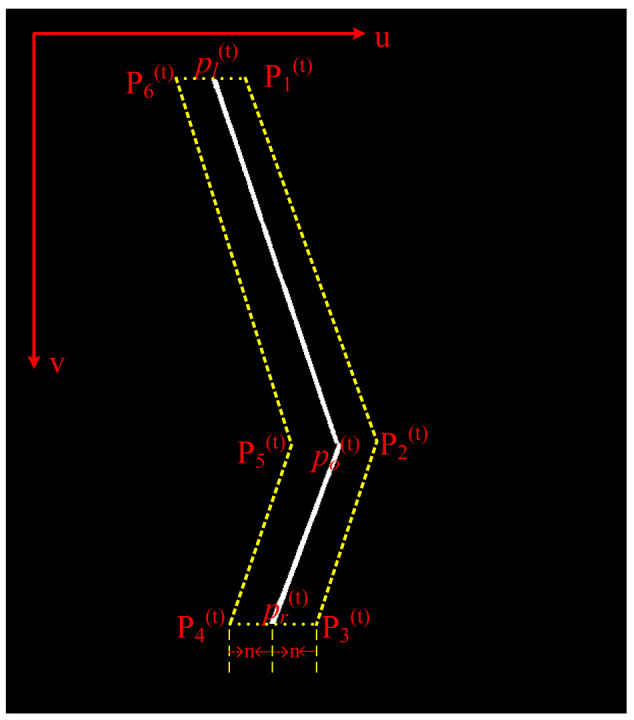
Dynamic ROI. *u* and *v* represent the pixel coordinate system. $\left\{p_{l}^{\left(t\right)},p_{o}^{\left(t\right)},p_{r}^{\left(t\right)}\right\}$ denotes three critical feature points of the laser stripe. ${\left\{P_{k}^{\left(t\right)}\right\}}_{k=1}^{6}$ denotes dynamic ROI.

Upon the determination of the three feature points, $\left\{p_{l}^{\left(t\right)},p_{o}^{\left(t\right)},p_{r}^{\left(t\right)}\right\}$, a polygonal confinement region encompassing the laser stripe can be algorithmically constructed, which is geometrically parameterized by the discrete point set ${\left\{P_{k}^{\left(t\right)}\right\}}_{k=1}^{6}$, as illustrated in Figure 3.

Assuming that the minimum separation distance between the polygonal boundary and laser centerline is 2n, the coordinates of the polygonal confinement region are defined as follows:

(4) $$\left\{\begin{array}{cc} P_{1}^{\left(t\right)} & =\left(p_{l}^{\left(t\right)}\left(u\right)+n,\;p_{l}^{\left(t\right)}\left(v\right)\right)\\ P_{2}^{\left(t\right)} & =\left(p_{o}^{\left(t\right)}\left(u\right)+n,\;p_{o}^{\left(t\right)}\left(v\right)\right)\\ P_{3}^{\left(t\right)} & =\left(p_{r}^{\left(t\right)}\left(u\right)+n,\;p_{r}^{\left(t\right)}\left(v\right)\right)\\ P_{4}^{\left(t\right)} & =\left(p_{r}^{\left(t\right)}\left(u\right)-n,\;p_{r}^{\left(t\right)}\left(v\right)\right)\\ P_{5}^{\left(t\right)} & =\left(p_{o}^{\left(t\right)}\left(u\right)-n,\;p_{o}^{\left(t\right)}\left(v\right)\right)\\ P_{6}^{\left(t\right)} & =\left(p_{l}^{\left(t\right)}\left(u\right)-n,\;p_{l}^{\left(t\right)}\left(v\right)\right) \end{array}\right.$$

Given the continuous image acquisition at 30 fps, the time interval between two consecutive frames is extremely short, leading to minimal changes between adjacent frames. Based on this observation, the ROI in the weld image for the current frame can be approximated as an irregular polygonal region from the previous frame. The core principle of the dynamic ROI is demonstrated in Algorithm 1 below.
**Algorithm 1** Core principle of dynamic ROI**Require:** Initial frame $I_{0}$ without noise
**Require:** Subsequent frames $\left\{I_{1},I_{2},...,I_{n}\right\}$
**Ensure:** Real-time laser stripe tracking

         **Initialization:**

    1: Extract initial ROI from $I_{0}$:

    2: ${\mathrm{ROI}}_{\mathrm{prev}}\leftarrow \mathrm{ProcessFrame}\left(I_{0}\right)$

         **Iterative Processing:**

    3: **for** each frame $I_{t}$ at time t≥1
**do**

    4:           Apply previous ROI:

    5:                 ${\mathrm{Cropped}}_{t}\leftarrow I_{t}\cap {\mathrm{ROI}}_{\mathrm{prev}}$

    6:           Extract new stripe region:

    7:                 ${\mathrm{Stripe}}_{t}\leftarrow \mathrm{DetectLaser}\left({\mathrm{Cropped}}_{t}\right)$

    8:           Propagate ROI to next frame:

    9:                 ${\mathrm{ROI}}_{\mathrm{prev}}\leftarrow \mathrm{CalculateNewROI}\left({\mathrm{Stripe}}_{t}\right)$

    10: **end for**


#### 2.2.2. Weld Seam Segmentation

The implementation of weld seam segmentation primarily involves three key steps: First, an adaptive threshold segmentation method is applied to binarize the weld seam image. Subsequently, morphological filtering is employed to eliminate isolated noise regions in the binary image. Finally, the laser stripe skeleton is extracted from the processed weld seam image to delineate the precise geometric profile.

##### Step 1: Thresholding

Accurate threshold determination is critical for achieving precise weld seam extraction in automated visual inspection systems. Traditional segmentation methods, such as fixed thresholding, often prove inadequate in welding applications due to dynamic challenges, including material heterogeneity across workpieces, intermittent arc glare interference, and a nonuniform spatter distribution. To address these limitations, this study implemented Otsu’s method [25], an adaptive thresholding method that statistically optimizes the inter-class separability. Let the weld seam segmentation candidate threshold be the grayscale value *T*. It divides the weld image into two classes: the foreground and background. The probabilities of the foreground and background classes are denoted as $p_{1}\left(T\right)$ and $p_{2}\left(T\right)$, respectively, while their corresponding mean grayscale values are represented by $\mu_{1}\left(T\right)$ and $\mu_{2}\left(T\right)$. The inter-class variance $\sigma_{b}^{2}\left(T\right)$ is then formulated as

(5) $$\sigma_{b}^{2}\left(T\right)=p_{1}\left(T\right)\;\cdot \;p_{2}\left(T\right)\;\cdot \;{\left[\mu_{1}\left(T\right)-\mu_{2}\left(T\right)\right]}^{2}$$

The optimal threshold $T^{*}$ is determined by exhaustively maximizing $\sigma_{b}^{2}\left(T\right)$ across all possible *T* values:

(6) $$T^{*}=\underset{0\leq T\leq L-1}{\arg\;\max}\left\{\sigma_{b}^{2}\left(T\right)\right\}$$

##### Step 2: Morphological Filtering

Due to the presence of spattering during the welding process, binary images of the weld seam often contain small isolated noise regions that require further filtering. We can introduce an area constraint to remove isolated regions with an area smaller than a specified threshold.

(7) $$R_{\mathrm{keep}}=\underset{k}{⋃}R_{k}\;\mathrm{where}\;\begin{array}{cccc} \; & R_{\mathrm{keep}} & \; & :\mathrm{Set}\;\mathrm{of}\;\mathrm{retained}\;\mathrm{regions}\;(\mathrm{in}\;\mathrm{pixels})\\ \; & R_{k} & \; & :k\mathrm{th}\;\mathrm{connected}\;\mathrm{component}\;(\mathrm{candidate}\;\mathrm{region})\\ \; & T_{\mathrm{area}} & \; & :\mathrm{Area}\;\mathrm{threshold}\\ \; & ⋃ & \; & :\mathrm{Union}\;\mathrm{operator}\;\mathrm{satisfying}\;\mathrm{Area}\left(R_{k}\right)\geq T_{\mathrm{area}} \end{array}$$

Simultaneously, by leveraging the slender nature of laser stripes, we can apply aspect ratio-based filtering constraints to further reduce the image noise while preserving the integrity of the laser stripes. Common binary image filtering methods include morphological filtering and spatial domain filtering. However, spatial domain filtering may blur the image edges while removing noise, which could affect subsequent analysis and processing. Therefore, we employed morphological filtering. Specifically, morphological opening not only effectively eliminates small noise points, protrusions, and burrs in the binary image but also preserves the integrity of the laser stripes well. The morphological opening operation is defined as follows:

(8) $$A\circ B=\left(A⊖B\right)⊕B$$

where *A* is the input image, *B* is the structuring element, ⊖ denotes the erosion operation, and ⊕ denotes the dilation operation in the morphology.

##### Step 3: Laser Stripe Centerline Extraction

Since laser stripes exhibit finite width distributions, the precise extraction of their centerlines is essential for accurately acquiring the weld seam feature points. The mainstream methods include the grayscale centroid method [26], Steger’s algorithm [27], and the medial axis transform (MAT) [28]. However, the grayscale centroid method demonstrates susceptibility to noise interference that may induce centerline deviations, while Steger’s algorithm incurs significantly higher computational complexity despite its superior precision. Consequently, this study adopted the MAT to achieve laser stripe centerline extraction with enhanced computational efficiency.

### 2.3. Optimized RANSAC Algorithm

**Figure 4:**
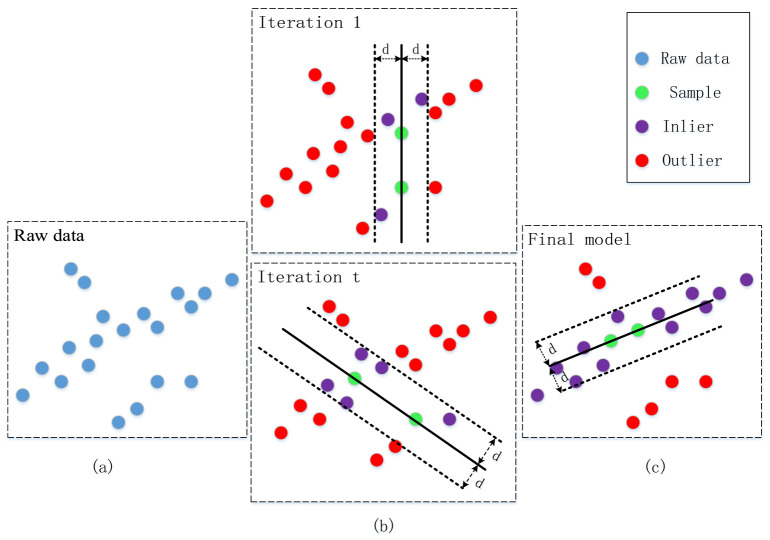
The principle of the RANSAC algorithm. (**a**) Raw Data Distribution (**b**) Iterative Optimization (**c**) Final Model Selection.

Due to effects from noise interference, the fitting process of the extracted laser stripe centerlines becomes necessary to construct high-precision weld seam models. The RANSAC algorithm [29], which effectively eliminates outliers during this procedure through an iterative mechanism, is illustrated in Figure 4.

According to the operational requirements of the RANSAC algorithm, weld seam model estimation requires a minimum sample size of 2. During the iterative hypothesis generation phase, two points are randomly selected from the point set to construct the initial laser stripe model.

(9) ax+by+c=0

As shown in Figure 4b, the iterative procedure comprises three key steps:

- Step 1: The computation of the Euclidean distance from all data points to the model M in each iteration.
  (10) $$d_{i}=\frac{\left|ax_{i}+by_{i}+c\right|}{\sqrt{a^{2}+b^{2}}}$$
  where $\left(x_{i},y_{i}\right)$ are the coordinates of the ith data point in the RANSAC algorithm.
- Step 2: The classification of inliers and outliers using a preset distance threshold, *w*. The parameter *w* is determined using the laser stripe width extracted from the initial reference frame. The discrimination criteria are as follows:
  (11) $$p_{i}=\left\{\begin{array}{cc} \mathrm{Inlier}, & d_{i}\leq w\\ \mathrm{Outlier}, & d_{i}>w \end{array}\right.$$
- Step 3: The selection of the model with the maximum number of inliers as the optimal laser stripe solution.

Although the RANSAC algorithm demonstrates satisfactory precision and robustness, its application to real-time weld seam extraction is constrained by two critical limitations:

(1) The stochastic sampling mechanism introduces inherent randomness and a trial-and-error nature, which necessitates excessive iterations and significantly degrades the computational efficiency;
(2) The predetermined fixed iteration count fails to dynamically adapt to varying inlier ratios, leading to a suboptimal trade-off between the processing speed and estimation accuracy.

To address the first limitation, we propose a slope-constrained sampling strategy based on temporal coherence. During continuous welding processes, adjacent laser stripes (frames *t* and t−1) exhibit minimal variation in their slope characteristics within the weld zone. This allows for the implementation of a temporal constraint:

(12) $$|\theta_{t}^{\left(i\right)}-\theta_{t-1}|\leq \theta_{\mathrm{th}}$$

where $\theta_{t}\left(i\right)$ denotes the slope of the *i*th sampled candidate in frame *t*, $\theta_{t-1}$ is the validated slope from frame t−1, and $\theta_{th}$ is the slope threshold used to verify the qualified point. These procedures effectively mitigate the aimlessness of random sampling while reducing the algorithm’s computational complexity.

To overcome the second limitation, a convergence criterion is activated when the improvement margin of the current optimal model falls below a predefined threshold, allowing for the early termination of redundant computational cycles. The optimized RANSAC algorithm is formally described in Algorithm 2.
**Algorithm 2** Improved slope-constrained RANSAC algorithm with early termination**Require:** Current frame laser stripe point set *P*, previous slope $m_{\mathrm{prev}}$,
    1:            max iterations *K*, distance threshold τ,
    2:            slope tolerance δ, consecutive patience *N*
**Ensure:** Optimal line model $\left(a^{*},b^{*}\right)$, inlier set $I^{*}$
    3: Initialize best inliers: $I^{*}\leftarrow ⌀$
    4: Initialize optimal model: $\left(a^{*},b^{*}\right)\leftarrow \left(0,0\right)$
    5: Iteration counter: k←0
    6: No improvement counter: no_improve←0
    7: **while** 
k<K
 **and** 
no_improve<N  
**do**
    8:     k←k+1
    9:     **repeat**
    10:            Randomly select two distinct points $p_{1}\left(x_{1},y_{1}\right),p_{2}\left(x_{2},y_{2}\right)\in P$
    11:            Compute slope: $m_{\mathrm{curr}}\leftarrow \frac{y_{2}-y_{1}}{x_{2}-x_{1}+\epsilon }$
    12:     **until** $|m_{\mathrm{curr}}-m_{\mathrm{prev}}|<\delta$
    13:     Construct line model: $a\leftarrow m_{\mathrm{curr}},\;b\leftarrow y_{1}-ax_{1}$
    14:     Temporary inlier set: I←⌀
    15:     **for** each point p(x,y)∈P **do**
    16:            Distance: $d\leftarrow \frac{\left|ax-y+b\right|}{\sqrt{a^{2}+1}}$
    17:            **if** d<τ **then**
    18:                I←I∪{p}
    19:            **end if**
    20:     **end for**
    21:     **if** $\left|I|>\right|I^{*}|$ **then**
    22:           $I^{*}\leftarrow I$
    23:            $\left(a^{*},b^{*}\right)\leftarrow \left(a,b\right)$
    24:            no_improve←0
    25:     **else**
    26:            no_improve←no_improve+1
    27:     **end if**
    28: **end while**
    29: **return** $\left(a^{*},b^{*}\right),I^{*}$


### 2.4. Weld Seam Feature Point Estimation

As illustrated in Figure 3, the laser stripe is partitioned into two distinct regions, $p_{l}^{\left(t\right)}p_{o}^{\left(t\right)}$ and $p_{o}^{\left(t\right)}p_{r}^{\left(t\right)}$, using $p_{o}^{\left(t\right)}$ as the demarcation point. By applying the optimized RANSAC algorithm within each partitioned region, two linear equations are derived as follows:

(13) $$\left\{\begin{array}{c} a_{1}x+b_{1}y+c_{1}=0\\ a_{2}x+b_{2}y+c_{2}=0 \end{array}\right.$$

The intersection point *X*, which serves as the welding seam feature point, is calculated as follows:

(14) $$X=A^{-1}C$$

where $A=\left[\begin{array}{cc} a_{1} & b_{1}\\ a_{2} & b_{2} \end{array}\right]$ and $C=\left[\begin{array}{c} -c_{1}\\ -c_{2} \end{array}\right]$.

### 2.5. Coordinate Transformation

For robotic welding trajectory generation, the laser image coordinates need to be transformed into robot base coordinates through three critical stages:

- Camera Calibration: Obtains the intrinsic matrix K and removes lens distortion.
- Laser Plane Calibration: Establishes the mapping from the 2D laser plane coordinates to the 3D camera space.
- Hand–Eye Calibration: Solves the transformation matrix $T_{E}^{C}$ between the robot tool end coordinate system {E} and the camera coordinate system {C}.

Following the transformation described in the preceding steps, the spatial coordinates of the weld seam in the robot base frame {B} can be derived through the following relationship:

(15) $$\left[\begin{array}{c} x_{B}\\ y_{B}\\ z_{B}\\ 1 \end{array}\right]=T_{B}^{E}\;\cdot \;T_{E}^{C}\;\cdot \;\left[\begin{array}{c} Z_{C}K^{-1}\left[\begin{array}{c} u\\ v\\ 1 \end{array}\right]\\ 1 \end{array}\right]$$

where $Z_{C}$ is the depth (Z-coordinate) of the weld seam feature point; $K^{-1}$ denotes the inverse of the camera intrinsic matrix; (u,v) are the pixel coordinates of the weld seam feature point; and $\left(x_{B},y_{B},z_{B}\right)$ are the spatial coordinates of the weld seam feature point in the robot base frame.

## 3. Experiments

### 3.1. Experimental Setup

**Figure 5:**
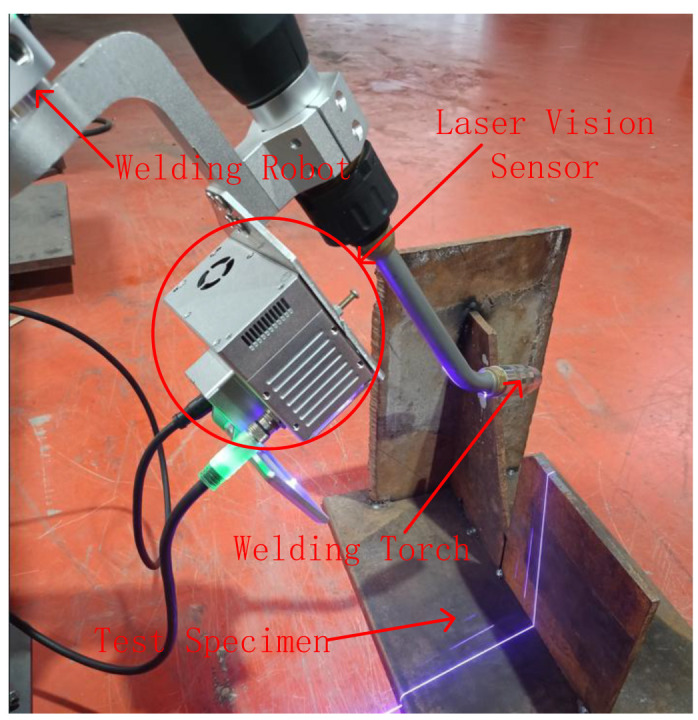
Experimental platform.

The experimental platform, as illustrated in Figure 5, consisted of a laser vision sensor mounted at the end effector of a 6-DOF collaborative robot. The laser vision sensor captured the weld images with laser stripes. The captured images were transmitted to the computer via TCP/IP communication. The computer then ran the proposed algorithm to extract the weld seam.

In this study, a 450 nm line laser was selected because the arc intensity in the neighborhood of the 450 nm band is relatively weak [10]. The experimental equipment parameters are summarized in Table 1.

### 3.2. Weld Seam Extraction Experiment

To evaluate the effectiveness of the proposed method, experimental trials were conducted with three representative weld configurations: fillet welds, butt welds, and lap joints. The welding parameters applied in the experiment are shown in Table 2.

**Figure 6:**
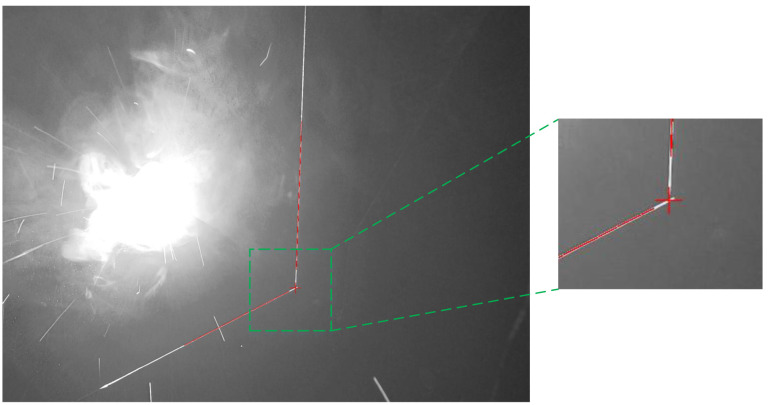
Fillet weld feature extraction results.

**Figure 7:**
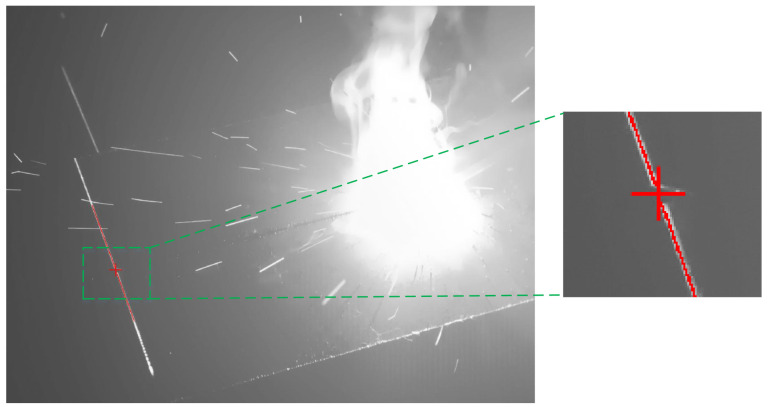
Butt weld feature extraction results.

**Figure 8:**
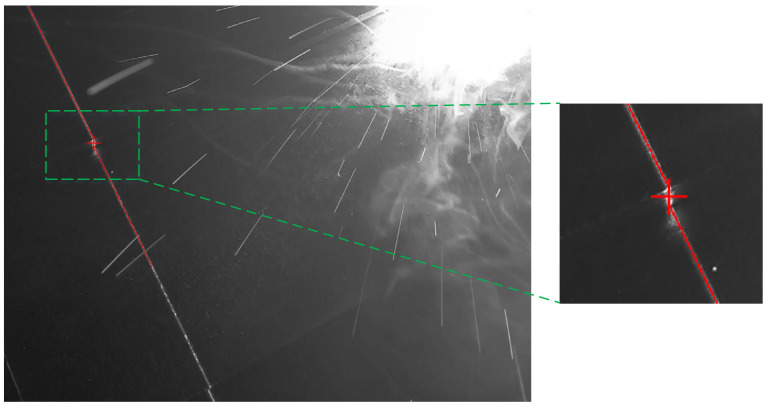
Lap weld feature extraction results.

A total of 172 fillet weld images, 157 lap joint weld images, and 131 butt joint weld images were acquired through the experimental process. The proposed algorithm achieved robust weld seam extraction across all experimental datasets. Representative experimental results are presented in Figure 6, Figure 7 and Figure 8. The red lines represent the algorithmically extracted laser stripes, while the red cross markers denote the identified weld seam locations. The right panel provides a magnified view of the extracted weld seam shown in the left image. As demonstrated in Figure 6, Figure 7 and Figure 8, the proposed algorithm achieved the high-precision extraction of fillet weld, butt weld, and lap weld features. These results confirm the method’s effectiveness in cross-type weld feature extraction across diverse joint configurations.

### 3.3. Ablation Experiment

#### 3.3.1. Impact of Dynamic ROI

The precise determination of the ROI serves as a critical preprocessing step in weld seam image extraction, where its accuracy fundamentally dictates the effectiveness of subsequent feature recognition. To validate the superiority of the dynamic ROI method, an image dataset comprising 172 fillet weld samples, 157 lap joint weld samples, and 131 butt weld samples was established. For each weld category, a dual-group controlled experiment was conducted: for the experimental group, we employed the proposed dynamic ROI extraction method, while for the control group, we utilized the conventional static ROI approach. During experimentation, both the processing time and extraction status (success/failure) were recorded for each sample. The average processing time [15,24,30] and extraction accuracy [13,19,30] for each algorithm across identical weld types were quantified as the performance metrics. The definition of the extraction accuracy was as follows:

(16) $$\mathrm{Accuracy}=\left(\frac{N_{\mathrm{correct}}}{N_{\mathrm{total}}}\right)\times 100\%$$

where $N_{\mathrm{correct}}$ denotes the number of images with correctly extracted welds, and $N_{\mathrm{total}}$ represents the total number of test samples. Comprehensive comparative results are presented in Table 3.

#### 3.3.2. Impact of Improved RANSAC Algorithm

To evaluate the impact of the improvements to the RANSAC algorithm on weld seam recognition, we conducted a comparative analysis using the execution time as the primary metric. The experimental dataset comprised 172 fillet weld images, 157 lap joint weld images, and 131 butt weld images, ensuring the comprehensive coverage of common welding scenarios. The results are presented in Table 4.

### 3.4. Comparative Experiments

**Figure 9:**
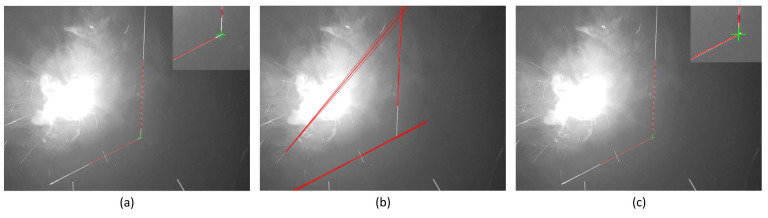
Feature extraction comparisons: (**a**) the proposed method successfully identified the actual weld seam characteristics; (**b**) the method from [15] failed to extract features from noise-contaminated images; (**c**) the method from [17] achieved effective feature extraction.

To evaluate the advantages of the proposed method, a comparative analysis was conducted with the approaches documented in [15,17]. Considering the prevalence of fillet welds in practical welding applications [12], the comparative experiments were specifically designed for this weld type. The experimental outcomes are demonstrated in Figure 9. The red lines represent the algorithmically extracted laser stripes, while the green cross markers denote the identified weld seam locations.

## 4. Discussion

### 4.1. Interference Mechanism of Spatter and Surface Reflections

In laser vision-based seam tracking systems, welding spatter and metal surface reflections degrade the laser stripe extraction accuracy through the following mechanisms:

- Spatter Interference: The dynamic dispersion of molten metal spatter induces the transient local occlusion and geometric distortion of the laser stripe, which may be misinterpreted as topological continuity features, leading to fragmented centerline reconstruction. Moreover, the high-velocity trajectories of spatter particles create spatio-temporal coupling interference with the laser stripe, significantly increasing the complexity of optical feature separation.
- Reflection Interference: Multi-order reflection artifacts generated by highly reflective metal surfaces produce competing optical signals, causing path ambiguity in stripe centerline extraction. Concurrently, photon saturation effects in specular reflection regions reduce the contrast threshold of valid signals, inducing subpixel-level edge resolution degradation.

### 4.2. Analysis of Dynamic ROI

As is quantitatively demonstrated in Table 3, the dynamic ROI method exhibited significant improvements over static ROI approaches in terms of both its extraction accuracy (averaging a 5.26% enhancement) and processing efficiency (averaging a 45.87% reduction in the computational time). This methodology focuses computational resources on localized laser stripe regions, thereby achieving dual technical benefits:

- The suppression of the arc-induced noise and spatter interference inherent to welding environments.
- A real-time processing capability at 30 fps.

The static ROI method employs a larger, predefined region to ensure the continuous coverage of the laser stripe throughout the welding process, while the dynamic ROI method adaptively tracks and confines the analysis to the immediate laser stripe area. Consequently, using a dynamic ROI achieves higher precision and improved computational efficiency relative to a static ROI, owing to its adaptive localization capability.

### 4.3. Analysis of Improved RANSAC Algorithm

As shown in Table 4, the experimental results demonstrate a significant increase in the convergence speed achieved by our enhanced RANSAC implementation. This improvement primarily stems from the introduced slope constraint mechanism, which effectively optimizes the random sampling process in the traditional RANSAC algorithm by reducing unnecessary iterations using prior geometric knowledge. Notably, the improved algorithm achieved 63% faster processing for fillet welds (21.26 ms → 7.97 ms) and maintained a speed increase of over 50%.

### 4.4. Analysis of Comparative Experiments

A quantitative evaluation of the computational efficiency and extraction accuracy was performed, with the statistical results being presented in Table 5.

Table 5 demonstrates that the proposed method achieved optimal performance in terms of both its detection accuracy and computational efficiency. The limitations of the method proposed in [15] were primarily caused by two aspects: (1) the absence of an ROI mechanism necessitated full-image preprocessing and weld detection using an improved Hough transform, resulting in an elevated computational load (21.55 ms), (2) and an insufficient adaptability to noise interference factors such as the welding spatter, smoke, and arc light led to significant accuracy degradation (84.88%). Although the method proposed in [17] enhanced noise immunity through Kalman filter-based laser stripe tracking (97.09%), it remained constrained by its rectangular ROI design and template matching strategy. In contrast, the proposed method employed an irregular polygonal ROI that further reduced the number of processed pixels compared to the rectangular ROI in [17], thereby achieving good real-time detection performance (7.97 ms) while maintaining high precision.

## 5. Conclusions

The accuracy of weld seam extraction is significantly challenged due to complex welding environments and interference factors such as the arc light and spatter generated during the process. To address these issues, this study proposed a laser vision-based weld seam extraction method incorporating a dynamic ROI mechanism and an improved RANSAC algorithm. The method dynamically generated an ROI for the current frame based on prior frame information, effectively suppressing noise interference while improving the computational efficiency. During image preprocessing, an adaptive threshold segmentation technique was employed to extract laser stripes. Subsequently, the RANSAC algorithm was optimized by refining the sampling strategies and dynamically adjusting the iteration counts, enabling subpixel-level precision in feature point extraction. Weld seam recognition experiments and comparative studies demonstrated the robust performance of the proposed method under complex working conditions, confirming its practical applicability. However, the current approach does not account for laser stripe interference caused by specular reflections on workpiece surfaces, the consideration of which will be prioritized in future research to enhance the segmentation robustness in such scenarios.

## Figures and Tables

**Table 1:** Technical specifications of experimental setup.

System Component	
Line laser	Wavelength: 450 nm; optical power: 80 mW; laser stripe width: 1 mm
Bandpass filter	(450 ± 20) nm
Imaging device	CCD camera (2592 × 1944 pixel) @ 30 fps
Computing unit	Intel Core i7-11800H @ 2.3 GHz, 16 GB RAM
Robot	Fairino 6-DOF robotic arm; repeatability: ±0.02 mm
Hand–eye calibration accuracy	1.2 mm

**Table 2:** Welding parameters for experimental configurations.

Parameter	Value
Welding process	GMAW
Welding materials	Q235 steel (8 mm thick)
Welding current	117 A
Welding voltage	22 V
Feed speed	3 mm/s
Welding speed	5 mm/s
Gas flow rate	18 L/min
Wire diameter	1.0 mm
Shielding gas	CO_2_

**Table 3:** Performance comparison between dynamic ROI and static ROI.

Method	Fillet Weld		Butt Weld		Lap Weld	
	Time (ms)	Acc. (%)	Time (ms)	Acc. (%)	Time (ms)	Acc. (%)
Dynamic ROI	7.97	98.84	5.31	96.18	6.26	97.45
Static ROI	12.40	91.28	10.20	92.37	13.59	94.27

**Table 4:** Execution time comparison between original and improved RANSAC algorithms.

Weld Type	Fillet Weld	Butt Weld	Lap Joint
Original RANSAC	21.26 ms	10.79 ms	12.63 ms
Improved RANSAC	7.97 ms	5.31 ms	6.26 ms

**Table 5:** Performance comparison of welding seam detection methods.

Method	Proposed	Wu et al. [15]	Li et al. [17]
Time (ms)	7.97	21.55	12.28
Accuracy (%)	98.84	84.88	97.09

## Data Availability

The data presented in this study are available upon request from the corresponding author.
